# Synbiotics in Oncology: A Scoping Review Protocol on Their Impact and Outcomes in Cancer Care

**DOI:** 10.3390/nursrep14020051

**Published:** 2024-03-22

**Authors:** Silvia Belloni, Cristina Arrigoni, Maria Helena Ceruso, Chiara Giacon, Arianna Magon, Gianluca Conte, Marco Alfredo Arcidiacono, Rosario Caruso

**Affiliations:** 1 Department of Gastroenterology, IRCCS Humanitas Research Hospital, 20089 Milan, Italy; silvia.belloni@humanitas.it; 2 Department of Public Health, Experimental and Forensic Medicine, Section of Hygiene, University of Pavia, 27100 Pavia, Italy; cristina.arrigoni@unipv.it; 3 Clinica San Michele, Savona, 17031 Albenga, Italy; mariahelena.ceruso01@universitadipavia.it; 4 IRCCS Policlinico San Matteo, 27100 Pavia, Italy; chiara.giacon01@universitadipavia.it; 5 Health Professions Research and Development Unit, IRCCS Policlinico San Donato, 20097 Milan, Italy; arianna.magon@grupposandonato.it (A.M.); gianluca.conte@grupposandonato.it (G.C.); 6 Medical Department, University Hospital of Parma, 43121 Parma, Italy; marcidiacono@ao.pr.it; 7 Department of Biomedical Sciences for Health, University of Milan, 20133 Milan, Italy

**Keywords:** probiotics, nutritional, metabolomics, cancer, clinical outcomes, symptoms, randomized controlled trials

## Abstract

Symptom management remains challenging in cancer care. Emerging from nutritional science, nutritional metabolomics has seen exponential growth over recent years, aiming to discern the relationship between dietary habits and health consequences. This protocol aims to present the rationale and methodology for conducting a scoping review to summarize the extent of evidence on synbiotics utilization in cancer symptom management among adults. The scoping review will be undertaken in accordance with the Joanna Briggs Institute (JBI) principles and the research process guided by the PRISMA 2020 scoping reviews extension. The following electronic databases will be searched from the inception: PubMed, Cinahl, Web of Science and Scopus. The authors expect to map the literature regarding the clinical outcomes, including patient-report measures and patient-experience measures, on which the effects of probiotics were tested, and identify potential gaps. This protocol presents a rigorous methodological approach to map the literature on the clinical outcomes that the utilization of synbiotics might improve. This analysis will shape future researchers to examine the efficacy of probiotics on specific clinical outcomes in oncology care. Nurses are uniquely positioned to influence cancer symptom management through the selection and use of appropriate interventions in the field of nutritional supplements, along with nutritional counseling.

## 1. Introduction

Addressing symptoms is effectively a critical aspect of cancer care [1,2]. The symptom burden of cancer patients has evolved due to longer survival and prolonged treatment options [3]. As a result of their diagnosis and treatment, cancer patients encounter a wide range of physical and psychological symptoms [1,4]. Emerging research and clinical observations indicate a wide variability in symptom experiences among oncology patients [4,5,6]. While some patients have relatively few symptoms, others undergoing the same treatment have several severe and distressing co-occurring symptoms [7,8]. Effective symptom management contributes to higher patient and family quality of life [9] and higher treatment adherence [10], and may even offer survival benefits [11].

In the field of cancer care, interventions rooted in electronic patient-reported outcomes (ePROs) or patient-reported outcome measures (PROMs) are gaining traction for symptom regulation [12,13,14]. Patient-reported outcomes (PROs) are reports directly from patients on their health status that have not been modified or interpreted by physicians or anyone else and are useful in providing optimal care [15]. PROMs are the tools used to measure PROs; these tools may be used to assess the patient’s health status, such as health-related quality of life [15]. PRO-based symptom management interventions typically consist of collecting patient symptom reports using ePRO tools and responding to these reports, which serve as measurement methods and digital interventions to monitor, detect, and manage patients’ symptoms, thereby improving their outcomes [16]. Recent evidence suggests that, compared to standard care, symptom management strategies based on ePRO could enhance the symptom burden, quality of life and overall survival of patients with cancer [17].

The domain of nutritional metabolomics, which delves into diet-associated molecular entities, has witnessed a surge in its application within cancer care, aiming to discern the nexus between dietary habits and health repercussions [18,19]. Chemotherapeutic interventions may disrupt intestinal microbiota composition, leading to dysbiosis, often associated with gastrointestinal and immune-related ailments [20]. Several strategies are in the pipeline to amend the microbiota, all grounded in the premise of fostering gut microbiota eubiosis. Introducing specific probiotic strains can potentially recalibrate the disrupted gut microbiota, positioning them as promising therapeutic avenues for addressing complications in cancer patients [21]. In particular, recent findings advocate the efficacy and safety of certain probiotic strains (e.g., *L. acidophilus*, *L. casei*, *B. longum*, or *L. rhamnosus*) as therapeutic agents against prevalent treatment-induced side effects in adult cancer patients, especially when administered either individually or in combinations over a span of at least 4 weeks [21].

A recent consensus statement from the International Scientific Association for Probiotics and Prebiotics (ISAPP) recommended utilizing the “synbiotics” definition in advancing research in the field of intestinal or extra-intestinal microbial ecosystems [22]. Synbiotics are defined as “a mixture comprising live microorganisms and substrate(s) selectively utilized by host microorganisms that confers a health benefit on the host” [22]. This definition encompasses probiotics and prebiotics (where each independently provides a health benefit), including two subsets of synbiotics: complementary (probiotic plus a prebiotic working independently) and synergistic (intended to act in combination, with the substrate being selectively used by the co-administered microorganism) [22].

Several experimental studies have yielded significant results substantiating the use of synbiotics in different cancer types (e.g., liver cancer, gastric cancer, lung cancer, breast cancer, brain cancer, and blood cancer) in the domain of cancer prevention, therapeutic intervention, and side effect management via various pathways (i.e., inhibition of NF-kB, reduced levels of H2AX, 8-hydroxy deoxyguanosine, RIG-I, downregulation of IL-17, and TNF signaling pathways) [23]. This evidence has been particularly corroborated in the field of breast cancer care [24]. Despite the experimental application of synbiotics in cancer care, a comprehensive summary of the literature to pinpoint specific cancer-related outcomes targeted by probiotics and to evaluate the extent of evidence supporting each outcome is noticeably absent [25]. This gap underscores the necessity of a structured approach to aggregate and scrutinize the existing research concerning the use of probiotics in mitigating cancer symptoms among adults. Consequently, this protocol delineates both the rationale and the methodology for a scoping review intended to systematically catalogue and evaluate the evidence on the role of synbiotics in cancer symptom management. Therefore, this review aims to lay a solid groundwork for subsequent in-depth research into how particular strains could effectively ease common symptoms and side effects associated with cancer treatment—factors crucially affecting patients’ quality of life and survival prospects. This protocol introduces a methodological framework to systematically map the literature on the clinical outcomes that the utilization of synbiotics might improve, providing a comprehensive overview that will guide future targeted research efforts.

## 2. Materials and Methods

### 2.1. Design and Review Question

This scoping review, adhering to the Joanna Briggs Institute (JBI) guidelines [26], seeks to determine: “What is the breadth and depth of evidence concerning the use of synbiotics in managing cancer symptoms in adults?” The chosen design aligns with the study’s aim to systematically map and assess a segment of the literature that has yet to be exhaustively explored, specifically focusing on the diverse clinical outcomes potentially enhanced by probiotic intake.

To further refine our exploration, we will address the following sub-question: In experimental research involving adult cancer patients, which specific cancer-related symptoms or clinical outcomes, including PROMs and PREMs, are identified as primary outcomes when synbiotics are employed as either complementary (add-on) or primary (stand-alone) therapeutic interventions? To ensure methodological quality in reporting, this review will follow the Preferred Reporting Items for Systematic Reviews and Meta-Analyses extension for Scoping Reviews (PRISMA-ScR) [27].

### 2.2. Type of Sources

This scoping review will include peer-reviewed scientific articles evaluating synbiotics’ efficacy in addressing cancer-related symptoms among adults. Accordingly, we will select papers including randomized controlled trials (RCTs) as a gold standard for assessing interventions’ efficacy and effectiveness. The research will not be limited to any language to find all the relevant publications. Additionally, we will delve into “grey” literature to access unpublished findings. To gain insights into emerging research, we will review conference proceedings, abstracts, and protocols of ongoing clinical trials listed in global medical registries.

### 2.3. Search Strategy

The following electronic databases will be searched from inception to the search date: PubMed, Cinahl, Web of Science and Scopus. The search strategy will be developed initially for the PubMed database, utilizing MESH headings and free-text words, and then it will be tailored for the other databases as appropriate. Key search terms will be combined with the Boolean operators “AND” and “OR” (Table S1). Hand-searching on Google Scholar and reference list-checking will be performed to identify additional studies. The WHO International Clinical Trials Registry and the Clinicaltrials.gov database will be searched to identify any unpublished ongoing original clinical trials with preliminary results. The abstracts of unpublished studies from some of the leading congresses in the medical area of various associations (American Society of Clinical Oncology (ASCO), European Society of Medical Oncology (ESMO), Multinational Association of Supportive Care in Cancer (MASCC)) will also be screened. The queries for systematically searching the databases will be developed following the Participants, Concept, and Context (PCC) framework [26].

### 2.4. PCC Framework

For participants, the focus is on adults aged 18 years and older who have been diagnosed with cancer. These individuals should have undergone treatments involving synbiotics, either as supplementary measures or as the primary course of action, during or after their cancer treatment. In terms of concept, the review will encompass a range of symptoms associated with cancer, the resulting clinical outcomes, and an array of PROMs and PREMs. Clinical trials will be considered from any context, whether home-based or clinical, and from any geographical location. Importantly, inclusivity is paramount; no distinctions will be made based on cultural, subcultural, ethnic, or gender identities.

### 2.5. Eligibility Criteria

Specific eligibility criteria have been established to determine the studies that will be considered for this scoping review. Studies that will be included align with the previously defined PCC framework. This implies they should primarily target adults aged 18 and older who have been diagnosed with cancer (any cancer diagnoses), delve into cancer-related symptoms and the treatment’s side effects, clinical outcomes, and both PROMs and PREMs, and be conducted in any clinical or home-based setting across all geographical regions. This means that we will consider any cancer-related symptom, treatment side effects and clinical outcomes reported by the patients. Additionally, the research design should be either experimental or quasi-experimental. A crucial aspect of the studies to be considered is the employment of synbiotics, according with ISAPP definition. These interventions can be used as the main treatment method or as a supplementary intervention in cancer care. On the other hand, studies that will be excluded from this review include those that focus on pediatric populations, purely observational studies without any experimental or quasi-experimental design (experimental design or randomized controlled trial without random assignment to treatment or control), and research that delves into interventions other than synbiotics, even in the realm of cancer care.

### 2.6. Studies Selection

The search results will be loaded into a reference manager software (Zotero) to remove duplicates. Two reviewers will independently screen all studies’ titles and abstracts after importing the final number of entries into an Excel sheet to find papers that may meet the eligibility criteria. Once all relevant studies have been selected, the two authors will collect full texts for the inclusion phase in the review. In case of missing information in the included articles, the reviewers will contact the studies’ authors. A third reviewer’s opinion will address disagreements between the two reviewers. The PRISMA flow diagram will document the selection process phase, with reasons for the articles’ exclusion [28].

### 2.7. Data Extraction

Following the completion of the screening procedure, two reviewers will design a piloted data extraction form for appraising and analyzing the body of evidence. The two reviewers will extract and enter data independently into the Excel spreadsheet data extraction form. During the first phase of data extraction, the form will be shaped based on the relevant information to guarantee that the data gathered will be relevant to the research question. The following data will be extracted from studies that encountered the selection criteria for inclusion: year of publication, country, study aim, study design, number of participants, cancer type and stage of treatment, intervention and control, duration of intervention, measurements, outcomes, and key findings. Additional variables will be considered as appropriate.

### 2.8. Data Synthesis and Presentation

The extracted results will be summarized and presented in the form of narrative synthesis, which will logically describe the aims of the included studies, the targeted populations, the intervention modalities, the outcomes and concepts, and the major findings. Should it be deemed suitable, the results may also be visualized as a structured “map” to represent the data. As the review progresses and a deeper comprehension of the study contents is achieved, these methodologies will be refined to ensure optimal presentation and understanding.

## 3. Discussion

This protocol describes the rationale and the methodological process for conducting a scoping review to map and identify gaps in the literature on cancer-related symptoms and clinical outcomes (including PROMs and PREMs) on which the effect of synbiotics was investigated. Patients with cancer encounter a wide range of potential treatment-related clinical outcomes that might represent a target for synbiotics’ mechanism of action. As nutritional metabolomics represents a new area of research in cancer care, a comprehensive systematic overview of cancer-related symptoms and clinical outcomes that might benefit from synbiotics use is pivotal to piloting future effectiveness research on the most relevant outcome in specific cancer populations. This step will be influential, considering that cancer symptom management remains complex, and an outcomes-driven approach could be strategically employed in determining the optimum treatment option.

Scoping reviews are an excellent method for determining the extent of a body of literature on a certain topic, as well as providing a clear indicator of the volume of literature available when it is unclear what further, more specific questions may be presented and valuably addressed by a more targeted systematic review [29]. Conversely, a systematic review and meta-analysis require specific selection criteria to address the research question, including targeted outcomes relevant to assessing clinical interventions’ effectiveness [30]. Outcome choice in a review is crucial, since it indicates the number of eligible studies that can be meta-analyzed and draws reliable conclusions regarding the investigated treatments [31]. In this regard, our scoping review will identify multiple cancer-related health outcomes, measured with validated scales, which could be improved by specific dietary cancer interventions based on nutritional metabolomics theory and mechanisms.

The practical implications of this research include future systematic reviews with meta-analyses on the effectiveness of certain probiotic strains with selected evidence-based outcomes in specific cancer populations. These systematic reviews will provide evidence-based strategies to guide clinical decision-making and the delivery of interventions for symptom management and policy development in the field of nutrition in cancer care [32]. Common side symptoms such as fatigue, diarrhea, vomiting, mucositis, or abdominal pain are distressing for patients undergoing cancer therapies. Thus, synbiotics could serve as a viable therapeutic option for treating and preventing these treatment-related effects as an add-on or stand-alone therapy [21].

### Limitations and Strengths

We foresee some potential limitations related to our scoping review. First, the methodological quality of the included studies will not be evaluated formally, as the goal of a scoping review is widely considered to provide an overview of available evidence regardless of its quality. It was considered that the evaluation process, followed by determining which papers to publish, had ensured the quality of the included papers. Second, our study attempts to answer a broader question on multiple outcomes, preventing the possibility of drawing conclusions on the efficacy and effectiveness of the investigated treatments. However, this aspect is consistent with the adopted methodology, which will help build future systematic reviews and meta-analyses on synbiotics’ effectiveness on targeted outcomes. Our study’s strength will be its adherence to a solid methodology. The protocol may be useful in providing transparency to the research process. Furthermore, the scoping review methodology assists in preventing the unnecessary duplication of research, and informs the scientific community about current research activities to answer a specific research question.

## 4. Conclusions

This protocol presents a rigorous methodological approach to map the literature on the clinical outcomes and cancer-related symptoms that the utilization of synbiotics might improve. This scoping review’s significant advantage is its ability to discern and highlight gaps and shortcomings in the current research landscape, offering clear pathways for subsequent investigations in this burgeoning field. The insights derived will shape and refine the focus of future studies, zeroing in on the impact of synbiotics on distinct clinical outcomes within oncology. Moreover, this review is poised to enrich the understanding of the significance of specific nutritional supplements in the context of cancer, ultimately guiding and enhancing dietary strategies for cancer nursing management.

## 5. Implications for Nursing Practice

This protocol proposes a rigorous empirical approach to mapping the literature on clinical outcomes and cancer-related symptoms that may be addressed by probiotics utilization. Nutritional activities are essential components of nursing in supporting cancer patients. Oncology nurses are pivotal in empowering patients in nutritional care by promoting healthy dietary interventions among cancer patients, boosting their role in providing dietary counseling throughout the disease course. Nurses are uniquely positioned to influence symptom management through recommendations on selecting and using appropriate interventions with nutritional supplements. In this regard, a mapping review protocol is essential to drive future multidisciplinary research on assessing the efficacy of synbiotics on targeted clinical outcomes along with related infectious complications. This enables nurses to empower patients, families, and caregivers to manage their own nutritional care and be aware of any potential complications.

## 6. Protocol Benefits and Application

- This protocol will serve as a methodological description of a scoping review on clinical outcomes that can be addressed by using synbiotics in oncology care;
- This accurate methodological description will guarantee rigor and transparency in the subsequent scoping review process;
- The current protocol will keep researchers updated on ongoing studies to avoid redundancy in the literature;
- The conduction of a scoping review on this topic will shape future systematic review research questions on specific clinical outcomes.

## Data Availability

The data presented in this study are available in the article and Supplementary Material.

## Supplementary Materials

The following supporting information can be downloaded at: https://www.mdpi.com/article/10.3390/nursrep14020051/s1, Table S1: Search strategy.

## Public Involvement Statement

No public involvement in any aspect of this research.

## Guidelines and Standards Statement

This manuscript was drafted against the Joanna Briggs Institute (JBI) guidelines and the Preferred Reporting Items for Systematic Reviews and Meta-Analyses extension for Scoping Reviews (PRISMA-ScR).
