# Supplementary material for: Synbiotics in Oncology: A Scoping Review Protocol on Their Impact and Outcomes in Cancer Care
Source: Nurs Rep. 2024 Mar 22;14(2):675–82. doi: 10.3390/nursrep14020051 (PMC10961746; doi:10.3390/nursrep14020051)
Supplement: Supplementary file 1 [file nursrep-14-00051-s001.zip › nursrep-2782389-supplementary.pdf]

Table S1. Search strategy

Database	Search query	Date of search	Results
**PubMed**	(("Neoplasms"[Mesh] OR Neoplas* OR Tumor* OR Tumour* OR Cancer* OR Carcino* OR Malignan* OR "Malignant Neoplasms" OR "Malignant Neoplasm" OR oncolog* OR oncog* OR Onkologie OR metasta* OR tumor cells OR tumour cells OR tumor cell OR tumour cell OR cancer cells OR cancer cell OR cell growth OR cancer survival OR cancer control OR advanced cancer OR tumorogen* OR tumorigen* OR tumorgen*) AND ("Probiotics"[Mesh] OR "Lactobacillus"[Mesh] OR "Bifidobacterium bifidum"[Mesh] OR "Lactobacillus casei"[Mesh] OR "Lactobacillus paracasei"[Mesh] OR "Lactobacillus rhamnosus"[Mesh] OR "Lactobacillus delbrueckii"[Mesh] OR "Lactobacillus brevis"[Mesh] OR "Lactobacillus johnsonii"[Mesh] OR "Lactobacillus plantarum"[Mesh] OR "Lactobacillus fermentum"[Mesh] OR "Lactobacillus acidophilus"[Mesh] OR "Lactobacillus plantarum"[Mesh] OR "Lactobacillus johnsonii"[Mesh] OR "Lactobacillus sakei"[Mesh] OR "Lactobacillus salivarius"[Mesh] OR "Streptococcus thermophilus"[Mesh] OR "Bifidobacterium longum subspecies infantis"[Mesh] OR "Bifidobacterium adolescentis"[Mesh] OR "Bifidobacterium animalis"[Mesh] OR "Bifidobacterium bifidum"[Mesh] OR "Bifidobacterium longum"[Mesh] OR "Bifidobacterium breve"[Mesh] OR "Saccharomyces boulardii"[Mesh] OR "Lactococcus lactis"[Mesh] OR "Lactobacillus delbrueckii subsp. bulgaricus"[tw] OR "Lactobacillus bulgaricus"[tw] OR "Bifidobacterium animalis subsp animalis"[tw] OR "Bifidobacterium animalis subsp lactis"[tw] OR "Lactococcus lactis subsp. lactis"[tw])) AND ((randomized controlled	June 2023	488

	trial[Publication Type] OR (randomized[Title/Abstract] AND controlled[Title/Abstract] AND trial[Title/Abstract])))		
**Cinahl**	((MH Neoplasms+) OR Neoplas* OR Tumor* OR Tumour* OR Cancer* OR Carcino* OR Malignan* OR "Malignant Neoplasms" OR "Malignant Neoplasm" OR oncolog* OR oncog* OR Onkologie OR metasta* OR "tumor cells" OR "tumour cells" OR "tumor cell" OR "tumour cell" OR "cancer cells" OR "cancer cell" OR "cell growth" OR "cancer survival" OR "cancer control" OR "advanced cancer" OR tumorogen* OR tumorigen* OR tumorgen*) AND ((MH Probiotics+) OR (MH Lactobacillus+) OR (MH "Bifidobacterium bifidum+") OR (MH "Lactobacillus casei+") OR (MH "Lactobacillus paracasei+") OR (MH "Lactobacillus rhamnosus+") OR (MH "Lactobacillus delbrueckii+") OR (MH "Lactobacillus brevis+") OR (MH "Lactobacillus johnsonii+") OR (MH "Lactobacillus plantarum+") OR (MH "Lactobacillus fermentum+") OR (MH "Lactobacillus acidophilus+") OR (MH "Lactobacillus plantarum+") OR (MH "Lactobacillus johnsonii+") OR (MH "Lactobacillus sakei+") OR (MH "Lactobacillus salivarius+") OR (MH "Streptococcus thermophilus+") OR (MH "Bifidobacterium longum subspecies infantis+") OR (MH "Bifidobacterium adolescentis+") OR (MH "Bifidobacterium animalis+") OR (MH "Bifidobacterium bifidum+") OR (MH "Bifidobacterium longum+") OR (MH "Bifidobacterium breve+") OR (MH "Saccharomyces boulardii+") OR (MH "Lactococcus lactis+") OR "Lactobacillus delbrueckii subsp. bulgaricus" OR "Lactobacillus bulgaricus" OR "Bifidobacterium animalis subsp animalis" OR "Bifidobacterium animalis subsp lactis" OR "Lactococcus lactis subsp. lactis") AND ((PT "randomized controlled trial") OR ((TI randomized OR AB randomized) AND (TI controlled OR AB controlled) AND (TI trial OR AB trial)))	June 2023	146

**Web of Science (WoS)**	ALL=((Neoplasms OR Neoplas* OR Tumor* OR Tumour* OR Cancer*) AND (Probiotics OR Lactobacillus OR "Bifidobacterium bifidum" OR "Lactobacillus casei" OR "Lactobacillus paracasei" OR "Lactobacillus rhamnosus" OR "Lactobacillus delbrueckii" OR "Lactobacillus brevis" OR "Lactobacillus johnsonii" OR "Lactobacillus plantarum" OR "Lactobacillus fermentum" OR "Lactobacillus acidophilus" OR "Lactobacillus plantarum" OR "Lactobacillus johnsonii" OR "Lactobacillus sakei" OR "Lactobacillus salivarius" OR "Streptococcus thermophilus" OR "Bifidobacterium longum subspecies infantis" OR "Bifidobacterium adolescentis" OR "Bifidobacterium animalis" OR "Bifidobacterium bifidum" OR "Bifidobacterium longum" OR "Bifidobacterium breve" OR "Saccharomyces boulardii" OR "Lactococcus lactis" OR "Lactobacillus delbrueckii subsp. bulgaricus" OR "Lactobacillus bulgaricus" OR "Bifidobacterium animalis subsp animalis" OR "Bifidobacterium animalis subsp lactis" OR "Lactococcus lactis subsp. lactis") AND ("randomized controlled trial" OR (randomized AND controlled AND trial)))	June 2023	443
**Scopus**	(INDEXTERMS (neoplasms) OR neoplas* OR tumor* OR tumour* OR cancer* OR carcino* OR malignan* OR "Malignant Neoplasms" OR "Malignant Neoplasm" OR oncolog* OR oncog* OR onkologie OR metasta* OR "tumor cells" OR "tumour cells" OR "tumor cell" OR "tumour cell" OR "cancer cells" OR "cancer cell" OR "cell growth" OR "cancer survival" OR "cancer control" OR "advanced cancer" OR tumorogen* OR tumorigen* OR tumorgen*) AND (INDEXTERMS (probiotics) OR INDEXTERMS (lactobacillus) OR INDEXTERMS ("Bifidobacterium bifidum") OR INDEXTERMS ("Lactobacillus casei") OR INDEXTERMS ("Lactobacillus paracasei") OR INDEXTERMS ("Lactobacillus rhamnosus") OR INDEXTERMS ("Lactobacillus delbrueckii") OR INDEXTERMS ("Lactobacillus	June 2023	1297

brevis") OR INDEXTERMS ("Lactobacillus johnsonii") OR INDEXTERMS ("Lactobacillus plantarum") OR INDEXTERMS ("Lactobacillus fermentum") OR INDEXTERMS ("Lactobacillus acidophilus") OR INDEXTERMS ("Lactobacillus plantarum") OR INDEXTERMS ("Lactobacillus johnsonii") OR INDEXTERMS ("Lactobacillus sakei") OR INDEXTERMS ("Lactobacillus salivarius") OR INDEXTERMS ("Streptococcus thermophilus") OR INDEXTERMS ("Bifidobacterium longum subspecies infantis") OR INDEXTERMS ("Bifidobacterium adolescentis") OR INDEXTERMS ("Bifidobacterium animalis") OR INDEXTERMS ("Bifidobacterium bifidum") OR INDEXTERMS ("Bifidobacterium longum") OR INDEXTERMS ("Bifidobacterium breve") OR INDEXTERMS ("Saccharomyces boulardii") OR INDEXTERMS ("Lactococcus lactis") OR TITLE-ABS-KEY ("Lactobacillus delbrueckii subsp. bulgaricus") OR TITLE-ABS-KEY ("Lactobacillus bulgaricus") OR TITLE-ABS-KEY ("Bifidobacterium animalis subsp animalis") OR TITLE-ABS-KEY ("Bifidobacterium animalis subsp lactis") OR TITLE-ABS-KEY ("Lactococcus lactis subsp. lactis")) AND (DOCTYPE ("randomized controlled trial") OR (TITLE-ABS (randomized) AND TITLE-ABS (controlled) AND TITLE-ABS (trial)))
